# Affections Liable to Occur to the Alveolar Process

**Published:** 1890-05

**Authors:** Geo. T. Thurer

**Affiliations:** U. of M.


### "Affections Liable to Occur to the Alveolar Process."

BY GEO. T. THURER, U. OF M.

The affections liable to occur to the alveolar process are quite numerous, and vary widely, according to the nature and permanency of the causes upon which such an affection depends.

One of the most important factors that are productive of pathological conditions of the alveolar process is a diseased condition of its lining membrane, the periosteum.

The alveolar processes, as well as the other osseous structures, are liable to necrosis or loss of vitality. When their connection with the periosteum—the source from which they derive all their nourishment and vitality—is destroyed death follows as a necessary consequence. The loss of vitality may be confined to the socket of a single tooth, but more frequently it extends to several and sometimes to the entire alveolar border. It may occur in either jaw, but is more liable to occur in the lower than the upper. When confined to the alveoli the dead part is never replaced with new bone, but examples are on record of the regeneration of a part and the whole of the lower jaw.

When one or more of the sockets of the teeth lose their vitality, nature exerts all her energies to separate the dead from the living bone. The process, called exfolliation, is supposed by some to consist of a sort of suppurative inflammation, but there is reason to believe it to be affected by the action of a corrosive fluid poured out from the fungus granulations of the living bone in contact with the necrosed part. The immediate cause of necrosis is the death of the periosteum, occasioned by inflammation. The cause of this, as has already been shown, is in a majority of cases, dental irritation.

Necrosis of the alveolar process occurs very frequently while the system is under the influence of mercurial medicines, and during billious and inflammatory fevers, and certain other constitutional diseases, as, syphilis, small-pox, scurvy, etc., and may also result from mechanical injuries.

Absorption or gradual destruction of the alveolar processes is always accompanied by a slight increase of redness, tumefaction, and a shrinking of the edges of the gum; but the diseased action here is so inconsiderable as to attract little attention, though upon close examination a slight discharge of purulent matter may be found.

The margins of the alveolar processes usually disappear as the destruction of the peridental membrane advances. The progress of this affection is quite slow and may go on for a number of years without affecting the stability of the teeth in their sockets.

Again in a considerable number of cases, especially those of the more chronic forms of disease, we may discover a definite thickening of the alveolar wall at or near its margin, which is clearly the result of exostosis brought about by irritation in the immediate neighborhood. In most if not all of these cases the peridental membrane will be found destroyed between this thickened rim and the root of the tooth, and if the gum be slit up and turned back, giving time for the blood to be sufficiently cleared away to get a good view of the parts, it is seen that the portion of the alveolus lying next to the teeth has been absorbed. We thus have an absorption of the inner portion of the alveolar wall and at the same time a deposit of bone on the outer portion which causes the gum tissue to be held away from the root of the tooth.

This usually occurs on the buccal and palatine wall, and as it causes the gum to project can be seen and may be felt with the finger. In these cases the absorption of the inner wall of the alveolus is readily determined by thrusting a delicate point through the tissues inside of this rim and exploring the widened alveolus. Even in slowly progressive cases this thickening of the alveolar rim is by no means constant, and then it is simply destroyed; in this case there is often a characteristic falling away of the gum, if the destructive process has eaten away the whole partition between two teeth. But many cases will be found where there is a deep pocket on the proximal side of the root of one tooth, while the membrane of its neighbor is injured; and in this case the gum will be supported in its position by a lamina of bone that will remain next to the sound membrane and appear complete. The thickening of the rim of the alveolus is usually very irregular, but very rarely fully encircles the tooth. It may border the destructive process in any position, and may sometimes be seen bordering a deep pocket over which the whole thickness of the alveolus is destroyed. And as the destructive process is going on beneath this thickening of the bone, there will often be found jagged prominences that will, in themselves, interfere with the healing process, if they are not removed at the beginning of the treatment. The destruction of bone in these cases seems to be a process of absorption, rather than necrosis, and is in part the result of pressure caused by the swelling of the membrane in its inflamed state and partly from the condition of irritation of the membrane to take on an absorptive action.

A tooth is sometimes, though rarely, slowly forced from its place by a deposit of bony matter in the bottom, or on the sides of the socket; more generally at the bottom. Two, or even three, teeth may be gradually displaced at the same time by exostosis of the alveoli, and this deposition usually proceeds so slowly that considerable time is required to effect a very perceptible change in the situation of the tooth or teeth affected. Irregularity may sometimes be produced in this way, especially when more than one socket is affected, and when this occurs with a person whose upper and lower teeth strike directly upon each other it occasions much inconvenience; for the elongated tooth must either be thrown from the circle of the other or by striking its antagonist prevent the jaws from coming together. The causes of this affection are not definitely understood. It is generally believed to be some irritation of the peridental membrane. But what causes the irritation is not well understood.

The susceptibility of the lining membrane to morbid impressions may sometimes be so great that the pressure of a very conical root may be sufficient to cause this effect; or, it may be produced by the pressure of a tooth that possesses only a very low degree of vitality.

But in connection with this class of cases must be taken another, in which absence of all pressure would seem to be the exciting cause of alveolar exostosis; as where a tooth has lost its antagonist tooth or teeth, and in consequence becomes elongated.

